# Are the physicochemical properties of antibacterial compounds really different from other drugs?

**DOI:** 10.1186/s13321-016-0143-5

**Published:** 2016-06-03

**Authors:** Jean-Paul Ebejer, Michael H. Charlton, Paul W. Finn

**Affiliations:** InhibOx Limited, Oxford Centre for Innovation, New Road, Oxford, OX1 1BY UK; Centre for Molecular Medicine and Biobanking, University of Malta, Msida, MSD 2080 Malta; University of Buckingham, Hunter Street, Buckingham, MK18 1EG UK

**Keywords:** Antibacterials, Molecular properties, Drug-like compounds, Cheminformatics database analysis, ChEMBL

## Abstract

**Background:**

It is now widely recognized that there is an urgent need for new antibacterial drugs, with novel mechanisms of action, to combat the rise of multi-drug resistant bacteria. However, few new compounds are reaching the market. Antibacterial drug discovery projects often succeed in identifying potent molecules in biochemical assays but have been beset by difficulties in obtaining antibacterial activity. A commonly held view, based on analysis of marketed antibacterial compounds, is that antibacterial drugs possess very different physicochemical properties to other drugs, and that this profile is required for antibacterial activity.

**Results:**

We have re-examined this issue by performing a cheminformatics analysis of the literature data available in the ChEMBL database. The physicochemical properties of compounds with a recorded activity in an antibacterial assay were calculated and compared to two other datasets extracted from ChEMBL, marketed antibacterials and drugs marketed for other therapeutic indications. The chemical class of the compounds and Gram-negative/Gram-positive profile were also investigated. This analysis shows that compounds with antibacterial activity have physicochemical property profiles very similar to other drug classes.

**Conclusions:**

The observation that many current antibacterial drugs lie in regions of physicochemical property space far from conventional small molecule therapeutics is correct. However, the inference that a compound must lie in one of these “outlier” regions in order to possess antibacterial activity is not supported by our analysis.

**Electronic supplementary material:**

The online version of this article (doi:10.1186/s13321-016-0143-5) contains supplementary material, which is available to authorized users.

**Graphical abstract:**
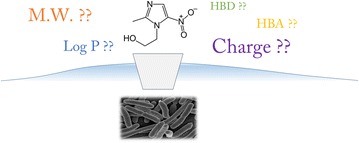
.

## Background

The continued development of antibiotic resistance in bacteria responsible for common infections is of growing worldwide concern. The Chief Medical Officer for England, Professor Dame Sally Davies has said that antimicrobial resistance poses a “catastrophic threat” to the health of the nation. It is estimated that in the countries of the European Union plus Iceland and Norway there were 25,000 deaths due to multi-drug resistant *Staphylococcus aureus*, *Enterococcus* spp., *Escherichia coli*, *Klebsiella* spp., *Enterobacter* spp. or *Pseudomonas aeruginosa* in 2007 [1]. However, this urgent medical need is not being adequately addressed by the development of new antibiotic therapies. Driven by a variety of issues, the scale of antibacterial research within the pharmaceutical industry has declined in recent years and the number of novel therapies approved has been very small [2].

The issue is made even more serious by the great technical difficulty of the discovery and development of antibacterial compounds. Large scale reviews of the antibacterial research at the pharmaceutical companies GSK [3], and, more recently, Astra Zeneca [4], have highlighted the challenges involved. Hit rates from high-throughput screens are often low. When hits are found, there is a high attrition rate in optimizing them to compounds with good levels of antibacterial activity, even when potent enzyme inhibitors are obtained.

One commonly cited rationalization of the failure of antibacterial drug discovery projects is lack of penetration to the site of action of the drugs. The bacterial cell wall presents a barrier to penetration, for which no analogous structure is present in mammalian cells. A corollary of this is that the physicochemical properties required for antibacterial activity may lie in a different region of property space to that of other drugs. Indeed, in the original Rule-of-5 publication [5] it was noted that antibacterial compounds were exceptions. In 2008 O’Shea and Moser [6] published a comparison of the physicochemical properties of 147 antibacterial compounds that were, at that time, currently used or under clinical investigation with a subset of 4623 non-antibacterial compounds from the commercially available CMC database. They found that antibacterial drugs occupy a remarkably different physicochemical property space—to take just two example properties, the mean molecular weight of the CMC data set was 338, whereas for Gram-positive compounds it was 813 and for Gram-negatives 414; mean calculated logD(7.4) was 1.6 for the CMC set, but −0.2 for Gram-positives and −2.8 for Gram-negatives. A more recent analysis by Davis et al. [7] based on a small dataset of 91 antibacterial and the 50 top-selling non-antibacterial marketed drugs, came to similar conclusions.

We were motivated to re-investigate the relationship between physicochemical properties and antibacterial activity for several reasons. The analyses of O’Shea and Moser and Davis et al. were based on a relatively small number of antibacterial compounds (necessarily) drawn from the limited number of classes of marketed antibiotics, many of which are natural products or their derivatives. Whilst the set of compounds analysed by O’Shea and Moser undoubtedly has the physicochemical property spectrum described, it is less clear that this spectrum is a requirement for antibacterial activity. Another interesting question is whether physicochemical properties vary by target class. A very recent analysis has looked at this question, again using a dataset of marketed drugs (157 antibacterials and 966 human) [8]. This analysis found a clear distinction between antibacterials targeting riboproteins, which were on average large and polar, and those with protein targets, which fell into classical drug-like ranges.

A disadvantage of using marketed drugs to study antibacterial-physicochemical property relationships is that, as the endpoints of drug development, they have been optimized to fulfil many criteria, of which antibacterial activity is only one. In recent years large cheminformatics databases, most notably ChEMBL [9], have been developed. An advantage of the use of ChEMBL data is that it allows the analysis of the significant quantity of biological assay data available in the literature. This is considerably more diverse, both in terms of biological target and chemotype, than the collection of marketed drugs and it potentially enables a more direct investigation of the physicochemical-activity relationships. In addition, the recent reports from Pharma companies of their antibacterial discovery efforts allow comparison with compounds arising from a pharmaceutical company compound collection.

Here we report our cheminformatics analysis of the small molecule antibacterial data available in ChEMBL. The analysis is sub-divided by compounds with activity (or inactivity) in biochemical and/or growth inhibition assays. Further analyses by chemotype and Gram-positive/negative classes have been performed. These datasets are compared with the set of marketed antibacterial drugs and a comparison set of marketed drugs taken from other therapeutic areas.

## Results and discussion

### Analysis of ChEMBL antibacterial activity data

ChEMBL (Version 20) contains over 10,000 compounds with at least one antibacterial activity data point. The detailed definition of activity is provided in Methods, but broadly corresponds to an MIC value of ≤8 µg/ml, which has commonly been used as an activity cutoff in the literature and in HTS campaigns. There are c. 2400 compounds with biochemical activity against a bacterial target protein. We have calculated physicochemical property distributions for the eight ChEMBL subsets described in Table 1. Briefly, these are: antibacterial actives (AA), compounds active in at least one antibacterial assay; antibacterial inactives (AI), compounds with only inactive antibacterial assay records; biochemical actives (BA), compounds active against at least one bacterial target protein; Biochemical Inactives (BI), compounds with only inactive biochemical assay records; Compounds with both biochemical and antibacterial activity (BAAA); Compounds active in biochemical assays but no reported antibacterial activity (BAAI); marketed antibacterial drugs (MAD); Marketed other, non-anticbacterial, drugs (MOD). One of the disadvantages of using marketed antibacterials for the determination of property requirements is their limited chemical diversity. As a measure of diversity we calculated the pairwise Tanimoto similarity, using ECFP fingerprints, of the AA, MAD and MOD sets (see Additional file 1: Figure S1). The pairwise Tanimoto distributions indicate limited diversity in the MAD set, with many highly similar pairs and a peak in the distribution at 0.475. The AA set has a lower peak (0.375) and very few highly similar pairs. As expected, the MOD distribution is the most diverse, with a peak at approximately 0.3. The greater diversity of the AA set (when compared to MAD) may allow a more general and widely applicable conclusion to be reached.

**Table 1:** Datasets used in the analysis

Description	Set label	Set size
Antibacterial actives compounds active in at least one antibacterial assay	AA	10,503
Antibacterial inactives, compounds with only inactive ChEMBL records	AI	340
Biochemical actives, compounds active against at least one antibacterial protein	BA	2470
Biochemical inactives, compounds with only inactive biochemical assay records	BI	399
Compounds active in both antibacterial and biochemical assays	BAAA	555
Compounds active in biochemical assays, but no reported antibacterial activity	BAAI	1835
Marketed antibacterial drugs	MAD	116
Marketed other (non-antibacterial) drugs	MOD	1225

**Fig. 1:**
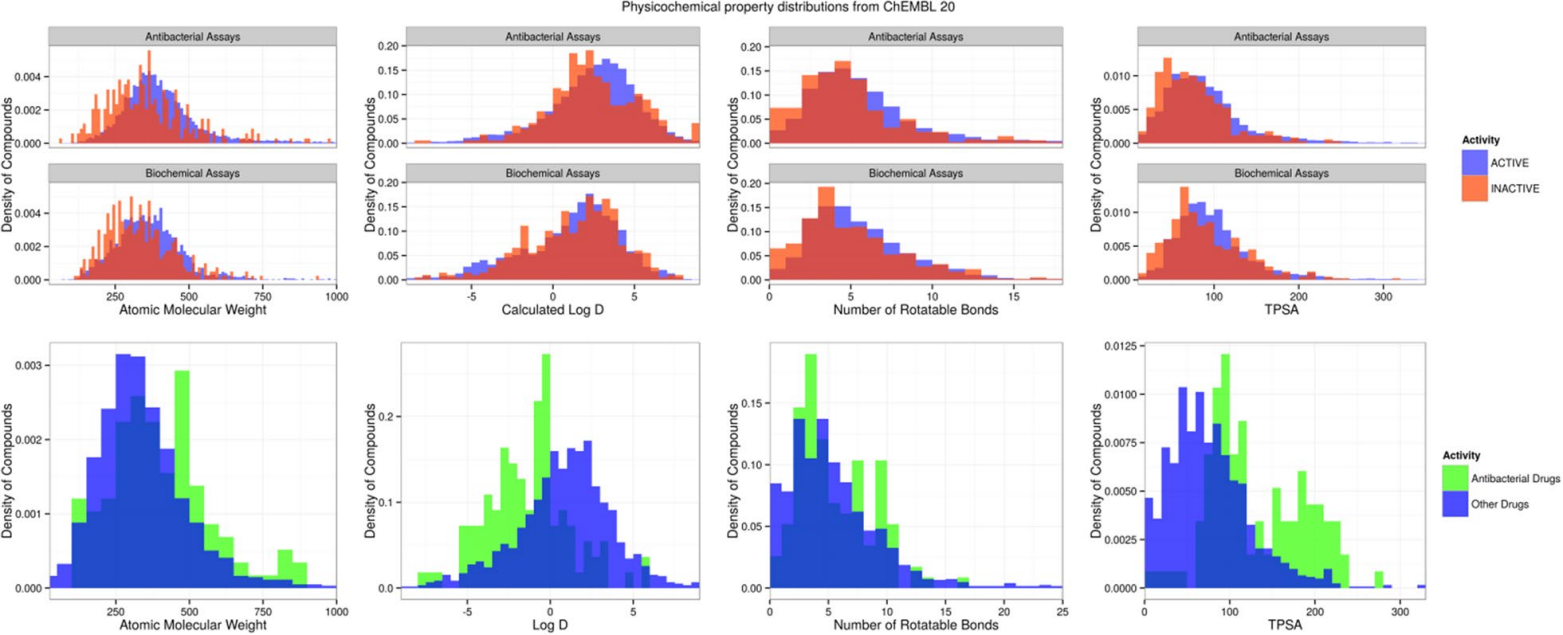
Distributions of molecular weight, calculated LogD, TPSA and number of rotatable bonds for antibacterial compounds from ChEMBL-20 (*upper panel*), marketed antibacterials and non-antibacterial marketed drugs (*lower panel*)

The distributions for nine physicochemical and related properties that describe the size, polarity, charge and flexibility of the subsets have been calculated. These are shown in Fig. 1, Table 2 and in Additional file 1, and are discussed below.

**Table 2:** Median values of physicochemical properties and charge class distribution

Set label	Median values								Charge classes			
	MWt	LogD	LogP	HBA	HBD	Rot bonds	Rings	TPSA	Acidic	Basic	Neutral	Zwitter
AA	385	2.8	3.2	6	2	5	3	81	1046	1746	5968	1743
AI	339	2.3	3.0	5	2	4	3	67	30	32	254	24
BA	358	1.7	2.2	6	2	4	3	92	546	290	1364	270
BI	321	1.7	1.9	6	2	4	3	80	59	58	253	29
BAAA	391	2.3	2.9	6	2	5	3	94	97	66	335	57
BAAI	346	1.4	1.7	6	2	4	3	93	441	209	988	197
MAD	389	−1.2	−0.7	8	3	4	3	117	37	27	23	29
MOD	316	1.1	1.8	5	2	4	3	69	195	425	495	110

#### Size

Average molecular weight is higher for the AA than the BA class, but not greatly so (385 vs. 358 Daltons). Active compounds (AA and BA) have a slightly higher molecular mass distribution than inactive ones (AI and BI), in keeping with the generally observed trend that adding molecular weight is associated with higher target affinity. The molecular weight distribution of the ChEMBL antibacterials is similar to marketed drugs. The perception that marketed antibacterials are larger than other drugs is confirmed in this analysis, as can be seen in the distributions in Fig. 1. Although the median values are not greatly different, the means show greater divergence, because of the large number of high molecular weight antibacterials (427 vs. 345 Da). Only 75 % of marketed antibacterials have molecular weight below 500 Da, whereas for marketed drugs the figure is 86.7 % for ChEMBL antibacterial actives 82.4 % and ChEMBL biochemical actives 90.3 %.

#### Polarity

Calculated LogD, calculated LogP, numbers of hydrogen bond donor and acceptors and TPSA distributions express aspects of compound polarity. Perhaps surprisingly, given the common perception that antibacterials tend to be highly polar, the distribution for these properties for the AA set largely fall within the classical Rule-of-5 regions. The bulk of antibacterial active compounds, 70.4 %, have calculated LogP values lying in the range 0–5, similar to the ranges seen in the BA and MOD sets (70.6 and 69.5 % respectively). Only 34.4 % of marketed antibacterials have logP in this range. The calculated LogD and calculated LogP distributions are rather similar, reflecting the fact that neutral compounds predominate for most of the classes. The other polarity-related properties tell a consistent story, with hydrogen-bond donor, hydrogen bond acceptor and TPSA distributions being similar across antibacterial, biochemical and marketed other drugs categories. Again marketed antibacterials are quite different: The percentage of compounds with TPSA less than 120 Å^2^ for the AA, BA and MOD sets is 81.1, 75.8 and 84.4 respectively, but only 52.6 % for marketed antibacterials.

Thus, in agreement with the previous analyses, the marketed antibacterial drugs do have a more polar distribution, with a lower median calculated logP, elevated donor and acceptor counts and higher TPSA (compare the MAD and MOD rows of Table 2). However, the ChEMBL analysis indicates that this is not a profile necessary to obtain antibacterial activity: the difference in profile between research compounds and marketed antibacterials could be driven by other factors. There are a limited number of classes of antibacterial drugs, each containing many structurally related compounds, including charge-carrying templates such as penicillins and fluoroquinolones. The physical property distributions are dominated by the relatively few chemotypes of the antibacterial drug classes that have made it to market, but that does not imply that high polarity is a necessary property of antibacterial compounds *per se*. Also, many antibacterial compounds have been developed for intravenous administration where high solubility is a key requirement for an efficacious compound. Thus the higher polarity of marketed antibacterials is likely to be influenced by optimization to obtain a suitable pharmacokinetic profile. For example, a review of industry DNA-gyrase/Topoisomerase IV programs over many decades [10] points to multiple occasions were the focus of optimization of the inhibitors was on increasing solubility to aid intravenous administration.

#### Charge

The antibacterial and biochemical datasets show a similar distribution with a preponderance of neutral molecules and approximately equal numbers of the other three classes. Although the non-systematic nature of the dataset precludes any firm conclusion, this is suggestive that overall charge state is not a significant influence on penetration. Interestingly, the profile of antibacterial drugs is very different, with a markedly lower number of neutral compounds so that all categories are well-represented, with a small preponderance of acidic compounds. The profile of the MOD set is intermediate, with the majority being neutral but with basic compounds also being highly represented.

#### Flexibility

None of the classes can be distinguished by the number of rotatable bonds or rings, and thus there is no indication of antibacterial compounds being any more or less flexible, on average, than other drugs.

Another comparison provides further evidence that antibacterial compounds are not atypical. If they were, then one would expect the properties of compounds that have biochemical activity and antibacterial activity “BAAA” to have a distinct profile from those that do not. Despite the size of the ChEMBL database, rather few compounds fall into this latter category. However, it is likely that most BA compounds without an antibacterial data record do not possess antibacterial activity and so we include these compounds in a putative (“BAAI”) set (see “Methods” for full definitions). These two sets, BAAA and BAAI, are not significantly different in their physical property profiles.

Another possibility that we investigated is whether there is an historical bias in the data. Drug discovery timelines are long and many of the marketed antibacterials were developed decades ago. It is possible that since the publication of the Rule-of-5 (1997), medicinal chemists working on antibacterial drug discovery projects shifted the physicochemical properties of the compounds they made from “antibacterial space” to “druglike space” in order to comply. There is no evidence for this. The molecular weight profile of compounds over time is remarkably constant, extending back to at least the mid 1970s (see Additional file 1: S2). The time series for logP (Additional file 1: S3) shows greater variability in the early years, but also has no evidence for any major shift over time.

### Gram-positive and Gram-negative active compound profiles

The cell envelopes of Gram-positive and Gram-negative bacteria are very different. The outer membrane of Gram-negative organisms presents an additional barrier to penetration, and there are many examples of compounds which are antibacterial against Gram-positive organisms but which are ineffective against Gram-negative ones. It is plausible that these differences in cell envelope structure would present differential barriers to penetration that would be reflected in divergent physicochemical property patterns for drugs effective against the two classes. Indeed, the O’Shea and Moser analysis indicated very different profiles for Gram-positive and Gram-negative antibacterials.

**Fig. 2:**
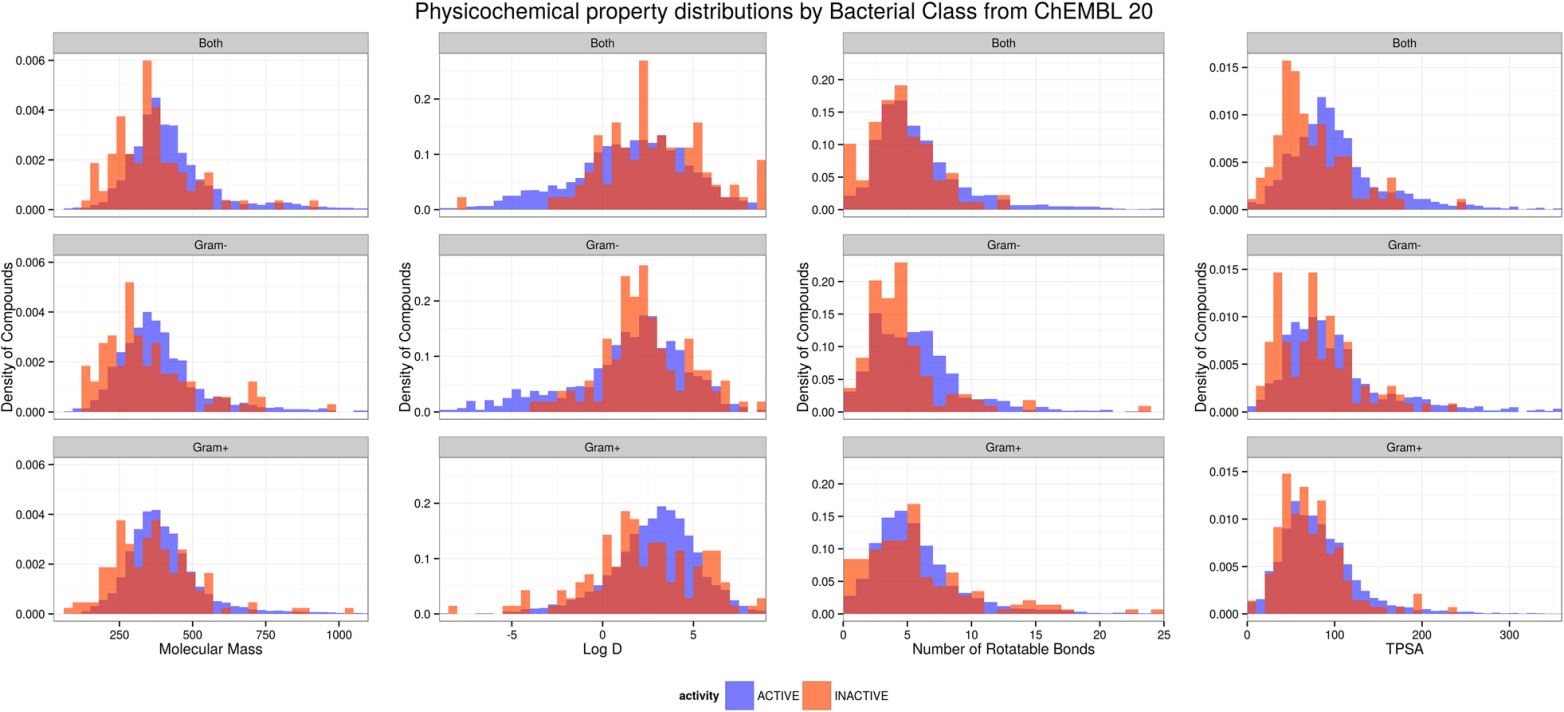
Comparison of distributions of molecular weight, calculated LogD, TPSA and number of rotatable bonds for Gram-negative and Gram-positive assay data in ChEMBL-20

However, the ChEMBL analysis does not support this conclusion. For all of the size, polarity and flexibility properties considered here, the differences between the distributions for Gram-positive and Gram-negative actives are unexpectedly small. Figure 2 shows the comparisons for molecular weight, calculated logD, TPSA and rotatable bonds; the distributions for the other properties are provided in Additional file 1: Figure S4.

The calculated logD values in the ChEMBL dataset show Gram-positive and Gram-negative active compounds having overlapping clogD ranges, with the Gram-positives having the higher mean and a slightly smaller standard deviation (3.1 ± 2.5 vs. 2.0 ± 3.4, respectively). The averages in both cases are more lipophilic than those obtained by O’Shea and Moser, with the ChEMBL analysis being closer to the MOD distribution. They are also consistent with the analyses of pharmaceutical company experiences (see below).

### Relationship to target class and site of action

Analysis of the physicochemical properties of antibacterials is further complicated by their varying sites of action. While some antibacterial classes have targets in the bacterial cytoplasm, others target the bacterial cell wall, in principle making access easier.

We have compared the physicochemical profiles of a variety of beta-lactam containing chemotypes, as cell-wall targeted agents, with fluoroquinolones and oxazolidinones as representatives of compounds hitting cytoplasmic targets. This represents a limited number of structural classes, because we have restricted the analysis to cases where at least 40 examples are present in ChEMBL.

This analysis shows limited differences between the two sets. The compounds with intracellular targets are slightly less polar (higher logD and slightly lower TSPA) but the differences are unlikely to be significant, especially as other classes of antibiotics with intracellular targets, such as the aminoglycosides, are very polar compounds.

### Comparison with literature analyses

Further support for the conclusion that antibacterial drugs often possess conventional drug-like physicochemical properties is provided by a comparison with the reports of pharmaceutical company experiences.

#### Polarity of Gram-positive and Gram-negative compounds

The results from the ChEMBL analysis are mirrored in the reports of the antibacterial drug discovery experience of pharmaceutical companies. Astra Zeneca (AZ) [4] reported that HTS compounds active against Gram-positive species had average clogD values that were slightly positive (range approximately 0.5–1.5) and thus higher than the Moser and O’Shea value of −0.2. For the Gram-positive phenotypic HTS campaign discussed in detail the average clogD was 3.6. The clogD range for Gram-negative organisms was slightly negative (range between −1.5 and 0); however, the median cLogD values reported for seven specified Gram-negative phenotypic screens ([4], Table 2) were between 1.7 and 3.5 The authors also report several specific examples of Gram-negative active compounds discovered by phenotypic screens with measured logD values between 1.4 and 4.3. Thus the logD ranges observed in the AZ examples are rather similar to the range of the ChEMBL AA compounds. Recently reported antibacterial pyrazolopyrimidinedione inhibitors of tRNA synthetases also have physicochemical properties in the typical drug-like ranges [11]. Taken together, these data indicate that compounds with physicochemical property values in the typical drug range can be potent antibacterials.

Antibacterial activity can be improved by increasing lipophilicity within these ranges, just as with mammalian cell assays. The DNA-gyrase review [10] includes several examples of improving Gram-positive activity with increasing lipophilicity, with several programs finding optimal logD in the 0–3 range. Davis et al. [7] showed that for a series of acylsulphonamide isoleucyl-tRNA inhibitors there is also a tendency for activity to improve with increasing lipophilicity, although the relationship is not simple and is organism dependent.

#### Charge state preferences for antibacterials

AZ analyzed the charge category of their antibacterial project actives relative to a representative sample of their internal compound collection. The collection as a whole was rather similar to the MOD profile presented here, but there was a lower proportion of neutral compounds in their Gram-positive antibacterial actives and a much lower proportion in the Gram-negative actives. The degree to which the AZ profiles reflect the particular set of targets that they have investigated historically is difficult to assess but it probably has some influence. They note, for example, that the large number of acidic compounds in their Gram-negative organism screens primarily belong to the β-lactam class.

The AZ analysis of the results obtained with efflux mutants is particularly interesting. Compounds with low efflux (assessed from the ratio of MIC against wild type and efflux mutant) tend to be small and polar or large and zwitterionic. These characteristics are similar to those associated historically with antibacterial activity, which perhaps indicates that the major issue is not penetration, but efflux.

## Conclusions

The set of currently approved antibacterial drugs do possess physicochemical property profiles that differ markedly from other drug classes, but this does not necessarily mean that such a profile is required for antibacterial activity. Indeed, although there are some differences, the physicochemical property distributions of antibacterial compounds in ChEMBL have rather similar profiles to other drug classes, with considerable overlap in the property ranges. An element of caution is needed in any broad cheminformatics study of this type, for example a proportion of the literature antibacterials could be achieving their effects by non-specific mechanisms such as membrane disruption and thus skewing the true distributions; however, given the size and diversity of the ChEMBL dataset, our cheminfomatics analysis demonstrates that the MAD physicochemical property profile is not a requirement and that “drug-like” physicochemical properties are compatible with antibacterial activity.

However, physicochemical properties in the optimal ranges are no guarantee of antibacterial activity. Our understanding of the physicochemical property-activity relationship remains rudimentary and provides little guidance for optimization of potent biochemical actives into antibacterial drugs. Following a detailed analysis of over 50 years of research and development directed against bacterial DNA gyrase/Topoisomerase IV [10] the authors conclude “At present, however, beyond imperfect correlations of logD and ionic charge with antibacterial potency in Gram-positive bacteria, there is disappointingly little further quantifiable and generalizable understanding of those specific factors that facilitate (or hinder) intracellular drug accumulation.”

Many compounds have better MICs against efflux pump mutant strains than against the wild type organism. Antibacterial classes with cytosolic target sites are also known to use active transport mechanisms to gain access to the cell (aminoglycosides and erythromycins are examples) and Trojan Horse inhibitors such as microcin C also use bacterial transporters to gain access to the cell, a mechanism that can also be exploited by some, but not all, synthetic analogues [12, 13]. The lack of predictive power of physicochemical properties alone is understandable if such mechanisms are common. The data are quite compatible with the hypothesis that access to the cytoplasm or periplasmic space rarely occurs by passive diffusion.

If this is the case, then there is a great need to learn more about the specific mechanisms of compound uptake. There is a growing understanding of the structural biology of the porins through which some drugs gain access to bacteria and of the relationship between porin structure and antibiotic transport. Additionally, next generation sequencing is providing new insights into the mechanisms of compound uptake that take a step towards the ability for rational design of antibacterial activity [14]. Most of the information we possess regarding penetration of compounds into bacteria comes from observing their antibacterial effects—we have very little data on the penetration of non-antibacterial compounds. Although technically demanding, further experimental data on the ability of organic compounds to enter the bacterial cytosol would be of great utility by providing a structurally more diverse dataset for computational studies.

These and other developments in the regulatory framework and funding environment may lead to improved success rates for antibacterial projects in the future [15]. This cheminformatics analysis represents another step towards an improved understanding and lays a foundation for further work.

## Methods

The analysis was carried out using the PostgreSQL version of ChEMBL V20. This was imported in a local installation of PostgreSQL (version 9.3) on Linux (Ubuntu 14.04). Compounds from antibacterial and biochemical assays were extracted using the parameters specified in Additional file 1: Tables S1 and S2 respectively. Each compound’s activity in the bioassay was labelled using ‘ACTIVE’, ‘SLIGHTLY_ACTIVE’ or ‘INACTIVE’. The categorization is not straightforward because of the large variety of activity units and the fact that these data are assembled from a great many individual sources, leading inevitably to a degree of uncertainty regarding experimental protocols and differences in the reported units (for example, µg/ml or µM). We have endeavoured to select cutoff values that are consistent and which correspond to typical values used for active/inactive decisions in drug discovery projects. The parameter filters for these three classes of activities are shown in Additional file 1: Tables S3, S4 and S5 respectively.

To perform the activity labelling, two matrices are constructed (one for biochemical assays and one for bacterial assays). Each matrix is labelled *m* and is defined as:

$$\begin{array}{*{20}c} {Activity = \left\{ {A,SA,I} \right\}} \\ {m_{ij} ::{\mathbb{N}} \times {\mathbb{N}} \to Activity} \\ \end{array}$$

Additional file 1: Table S6 describes the parameter filters used to define compounds which have been approved as drugs into two distinct subsets; antibacterials (labelled MAD) and non-antibacterials (labelled MOD).

### Definition of compound sets

**Fig. 3:**
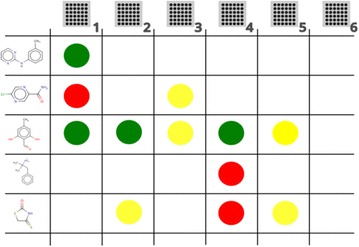
Assays represented as columns and compounds represented as rows with activities in the intersection of the two. Four activity states are represented: Not tested (void cell), inactive (*red*), slightly active (*yellow*) and active (*green*). A single ChEMBL compound may be present in multiple assays (e.g. both biochemical and antibacterial)

A schematic of the antibacterial structure–activity relationship matrix for ChEMBL used in our analysis is shown in Fig. 3. Each compound is represented as a vector of activities, e.g. the third compound in Fig. 3 may be represented as:

$${\text{c}}_{3} = \left[ {{\text{ A}},{\text{A}},{\text{SA}},{\text{A}},{\text{SA}}, - , - } \right]$$

We define activity of molecules over two cases – the antibacterial assays (denoted AA) and the biochemical assays (denoted BA). For compounds tested in AA and BA, an active is defined as:

$$Active\left( {c_{j} } \right): = \exists i:m_{ij} = A \wedge {\nexists }i^{'} :m_{{i^{{{\prime}}} j}} = I$$

For compounds tested in AA and BA, an inactive is defined as:

$$Inactive\left( {c_{j} } \right): = \exists i:m_{ij} = I \wedge {\nexists }i^{{\prime }} :m_{{i^{{^{{\prime }} }} j}} \ne I$$

The set of compounds which are both active in AA and BA is defined as:

$$AABA: = \left\{ {x:x \in AA \wedge Active\left( x \right)} \right\} \cap \left\{ {y:y \in BA \wedge Active\left( y \right)} \right\}$$

The set of compounds which are active in BA and inactive in AA, was originally defined as:

$$AABI: = \left\{ {x:x \in AA \wedge Inactive\left( x \right)} \right\} \cap \left\{ {y:y \in BA \wedge Active\left( y \right)} \right\}$$

However this returned very few compounds, so we relaxed this to the following definition:

$$AABI: = \left\{ {x:x \notin AA \wedge x \in BA \wedge Active\left( x \right)} \right\}$$

These rules give us the active and inactive compound sets for AA and BA. We have compounds that are active in both AA and BA (labelled AABA) and compounds which are active in BA, but have no activity recorded in AA (labelled BAAI). In combination with the MAD and MOD sets, this provides six sets in total.

Each of these six sets is clustered to avoid having similar molecules in the set, and to avoid chemotype bias [16]. For each compound set Morgan (ECFP like) fingerprints of length 2048 bits with diameter 4 were calculated, using RDKit (release 2014_09_2). These were then clustered using the SUBSET program [17] with a similarity threshold of 0.65. This guarantees that any pair of molecules has a Tanimoto similarity of at least 0.65. Note that the SUBSET algorithm is non-deterministic.

A three step standardization procedure was performed before calculating the physicochemical property distributions. Step one performs a standardization to ensure a consistent representation of, for example, aromatic rings and nitro groups. As the second step, where molecules have multiple components, salts are removed using a list of known salts. If there are still multiple components remaining after salt stripping, the largest component is retained. In the final step, the ionization state of the compounds is set to the most prevalent form at pH 7.4, based upon a set of substructure rules. These physicochemical properties and the methods of calculation for the molecular sets are given in Additional file 1: Table S7.

## Additional file

10.1186/s13321-016-0143-5 **Table S1.** Filters used to extract compounds from Antibacterial assays. **Table S2.** Filters used to extract compounds from Biochemical assays. **Table S3a.** Filters used to define antibacterial and biochemical compounds as ACTIVE. **Table S3b.** Additional filters (only one is applied per activity) used to define antibacterial and biochemical compounds as ACTIVE. **Table S4a.** Filters used to define antibacterial and biochemical compounds as SLIGHTLY_ACTIVE. **Table S4b.** Additional filters on the 'activities' table used to define antibacterial and biochemical compounds as SLIGHTLY_ACTIVE. **Table S5.** Filters used to define antibacterial and biochemical compounds as INACTIVE. **Table S6.** Filters used to extract compounds which have been approved as drugs. **Table S7.** Computed Descriptors for Dataset. **Figure S1.** Pairwise Tanimoto similarity of the MAD (a), AA (b) and MOD (c)sets. These distributions were calculated on the full sets, prior to clustering. **Figure S2.** Boxplot for time series of molecular weight of antibacterial active compounds in the ChEMBL dataset. **Figure S3.** Boxplot for time series of calculated logP of antibacterial active compounds in the ChEMBL dataset. **Figure S4.** Property distribution comparisons for Gram negative and Gram positive active compounds.
