# Supplementary material for: Are the physicochemical properties of antibacterial compounds really different from other drugs?
Source: J Cheminform. 2016 Jun 3;8:30. doi: 10.1186/s13321-016-0143-5 (PMC4891840; doi:10.1186/s13321-016-0143-5)

Supplementary Information

Table S1: Filters used to extract compounds from Antibacterial assays

ChEMBL Table	Column	Value for Filtering
assays	bao_format	= 'BAO_0000218'
assays	assay_type	= 'F'
assays	confidence_score	= 1 **or** 2
organism_class	l1	= 'Bacteria'
organism_class	l2	= 'Gram-Positive' **or** 'Gram-Negative'
molecule_dictionary	molecule_type	= 'Small molecule'
molecule_dictionary	polymer_flag	= 0
activities	data_validity_comment	= [Null] **or** 'Manually validated'
activities	standard_flag	= 1
activities	potential_duplicate	<> 1

Table S2: Filters used to extract compounds from Biochemical assays

ChEMBL Table	Column	Value for Filtering
assays	bao_format	= 'BAO_0000217' **or** 'BAO_0000224' **or** 'BAO_0000357' **or** 'BAO_0000223'
assays	assay_type	= 'B'
assays	confidence_score	>= 6
organism_class	l1	= 'Bacteria'
organism_class	l2	= 'Gram-Positive' **or** 'Gram-Negative'
molecule_dictionary	molecule_type	= 'Small molecule'
molecule_dictionary	polymer_flag	= 0
activities	data_validity_comment	= [Null] **or** 'Manually validated'
activities	standard_flag	= 1
activities	potential_duplicate	<> 1

Table S3a: Filters used to define antibacterial and biochemical compounds as ACTIVE

ChEMBL Table	Column	Value for Filtering	Comment
activities	pchembl_value	= [Null] **or** >= 4.75	
activities	standard_value	> 0	

Table S3b: Additional filters (only one is applied per activity) used to define antibacterial and biochemical compounds as ACTIVE

standard_type	standard_units[1]	standard_value		standard_relation
=	=	**Lower bound** >=	**Upper Bound** <=	(One of the following four operators)
'Inhibition'	'%'	70	100	>,=,>=,~
'IC50'	'nm'		25,000	<,=,<=,~
'IC50'	'ug.ml-1' or 'ug ml-1'		8	<,=,<=,~
'IC90'	'nm'		100,000	<,=,<=,~
'IC90'	'ug.ml-1' or 'ug ml-1'		32	<,=,<=,~
'MIC'	'nm'		25,000	<,=,<=,~
'MIC'	'ug.ml-1' or 'ug ml-1'		8	<,=,<=,~
'MIC50'	'nm'		25,000	<,=,<=,~
'MIC50'	'ug.ml-1' or 'ug ml-1'		8	<,=,<=,~
'MIC95'	'um'		100	<,=,<=,~
'MIC95'	'ug.ml-1' or 'ug ml-1'		32	<,=,<=,~
'EC50'	'nm'		25,000	<,=,<=,~
'EC50'	'ug.ml-1' or 'ug ml-1'		8	<,=,<=,~
'ED50'	'nm'		25,000	<,=,<=,~
'ED50'	'mg.kg-1'		250	<,=,<=,~
'MBC'	'ug.ml-1' or 'ug ml-1'		8	<,=,<=,~
'MBC90'	'ug.ml-1' or 'ug ml-1'		32	<,=,<=,~
'GI'	'%'	70	100	>,=,>=,~
'GI'	'nm'		25,000	<,=,<=,~
'GI'	'um'		25	<,=,<=,~
'GI50'	'nm'		25,000	<,=,<=,~
'GI50'	'ug.ml-1' or 'ug ml-1'		8	<,=,<=,~
'Kd'	'nm'		25,000	<,=,<=,~
'Ki'	'nm'		25,000	<,=,<=,~

[1]Converted to lowercase to normalize the units' text. Note that some standard types only make sense for a particular assay context (e.g. Kd for biochemical assays, MBC for antibacterial assays).

Table S4a: Filters used to define antibacterial and biochemical compounds as SLIGHTLY_ACTIVE

ChEMBL Table	Column	Value for Filtering	Comment
activities	standard_value	> 0	

Table S4b: Additional filters on the 'activities' table used to define antibacterial and biochemical compounds as SLIGHTLY_ACTIVE.

standard_type	standard_units[1]	standard_value		standard_relation
=	=	**Lower bound** >	**Upper Bound** <	(One of the following four operators)
'Inhibition'	'%'		70	<,=,<=,~
'IC50'	'nm'	25,000		>,=,>=,~
'IC50'	'ug.ml-1' or 'ug ml-1'	8		>,=,>=,~
'IC90'	'nm'	100,000		>,=,>=,~
'IC90'	'ug.ml-1' or 'ug ml-1'	32		>,=,>=,~
'MIC'	'nm'	25,000		>,=,>=,~
'MIC'	'ug.ml-1' or 'ug ml-1'	8		>,=,>=,~
'MIC50'	'nm'	25,000		>,=,>=,~
'MIC50'	'ug.ml-1' or 'ug ml-1'	8		>,=,>=,~
'MIC95'	'um'	100		>,=,>=,~
'MIC95'	'ug.ml-1' or 'ug ml-1'	32		>,=,>=,~
'EC50'	'nm'	25,000		>,=,>=,~
'EC50'	'ug.ml-1' or 'ug ml-1'	8		>,=,>=,~
'ED50'	'nm'	25,000		>,=,>=,~
'ED50'	'mg.kg-1'	250		>,=,>=,~
'MBC'	'ug.ml-1' or 'ug ml-1'	8		>,=,>=,~
'MBC90'	'ug.ml-1' or 'ug ml-1'	32		>,=,>=,~
'GI'	'%'		70	<,=,<=,~
'GI'	'nm'	25,000		>,=,>=,~
'GI'	'um'		25	>,=,>=,~
'GI50'	'nm'	25,000		>,=,>=,~
'GI50'	'ug.ml-1' or 'ug ml-1'	8		>,=,>=,~
'Kd'	'nm'	25,000		>,=,>=,~
'Ki'	'nm'	25,000		>,=,>=,~

Note that these are mostly the thresholds used for the ACTIVE label and multiplied by a factor of four.
[1]Converted to lowercase to normalize the units' text. Note that some standard types only make sense for a particular assay context (e.g. Kd for biochemical assays, MBC for antibacterial assays)

Table S5: Filters used to define antibacterial and biochemical compounds as INACTIVE. [1]Converted to lowercase to normalize the comment.

ChEMBL Table	Column	Value for Filtering	Comment
activities	standard_type	= 'Inhibition' **or**<br>= 'IC50' **or**<br>= 'IC90' **or**<br>= 'MIC' **or**<br>= 'MIC50' **or**<br>= 'MIC95' **or**<br>= 'EC50' **or**<br>= 'ED50' **or**<br>= 'MBC' **or**<br>= 'MBC90' **or**<br>= 'GI' **or**<br>= 'GI50' **or**<br>= 'Kd' **or**<br>= 'Ki'	
activities	activity_comment[1]	<> 'active' **and**<br><> 'not tested' **and**<br><> 'not done' **and**<br><> 'no data' **and**<br><> 'not determined' **and**<br><> 'not reported' **and**<br><> 'not evaluated' **and**<br><> 'inconclusive'	
activities	standard_value / activity_comment[1]	standard_value = [NULL] **or** standard_value = 0 **or**<br>activity_comment = 'not active' **or**<br>activity_comment = 'inactive' **or**<br>activity_comment = 'no activity'	

Table S6: Filters used to extract compounds which have been approved as drugs.

ChEMBL Table	Column	Value for Filtering
molecule_dictionary	molecule_type	= 'Small molecule'
molecule_dictionary	polymer_flag	= 0
molecule_dictionary	max_phase	= 4
molecule_dictionary	indication_class[1]	Like<br>'%anti-bacterial%'<br>'%antibacterial%'

[1]Converted to lowercase to normalize the comment.

Table S7: Computed Descriptors for Dataset

Descriptor	Notes
Atomic molecular mass	Computed using the PostgreSQL database cartridge in RDKit
Molecular log P	Computed using the PostgreSQL database cartridge in RDKit
Log D	Imported from ChEMBL Compound_Properties (column acd_logd)
Number of hydrogen bond acceptors	Computed using the PostgreSQL database cartridge in RDKit
Number of hydrogen bond donors	Computed using the PostgreSQL database cartridge in RDKit
Number of atoms	Computed using the PostgreSQL database cartridge in RDKit
Number of heavy atoms	Computed using the PostgreSQL database cartridge in RDKit
Number of hetero atoms	Computed using the PostgreSQL database cartridge in RDKit
Number of rotatable bonds	Computed using the PostgreSQL database cartridge in RDKit
Number of rings	Computed using the PostgreSQL database cartridge in RDKit
Number of aromatic rings	Computed using the PostgreSQL database cartridge in RDKit
TPSA	Computed using the PostgreSQL database cartridge in RDKit
Total negative charge	Total negative charges on molecule
Total positive charge	Total positive charges on molecule
Atom negative charge	Number of atoms with a negative charge
Atom positive charge	Number of atoms with a positive charge
Atom charge count	Number of charged atoms
Charge class	ZWIT: has both negative and positive charges,<br>POS: has only positive charges,<br>NEG: has only negative charges,<br>NEUT: has no charges
Bacterial class	GRAM+: if compound is active in AA against GRAM+ target classes, and there are no active records against GRAM- classes.<br>GRAM-: if compound is active in AA against GRAM- target classes, and there are no active records against GRAM+ classes.<br>BOTH: if compound is active in AA against both GRAM- and GRAM+ bacterial classes.

Figure S1. Pairwise Tanimoto similarity of the MAD (a), AA (b) and MOD (c)sets. These distributions were calculated on the full sets, prior to clustering.

a)

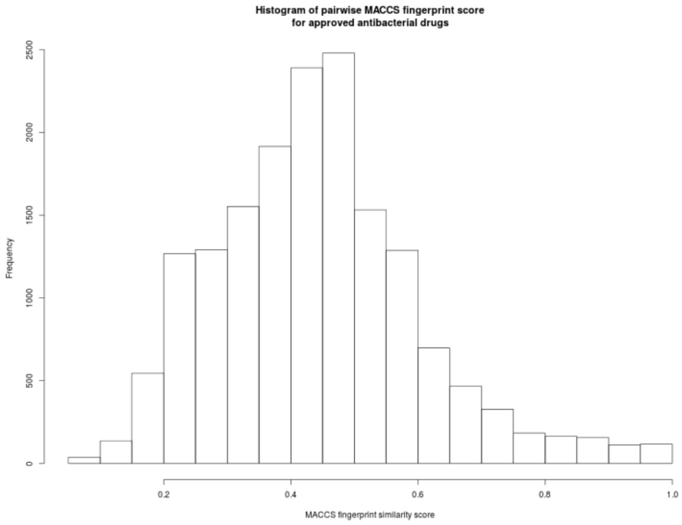

b)

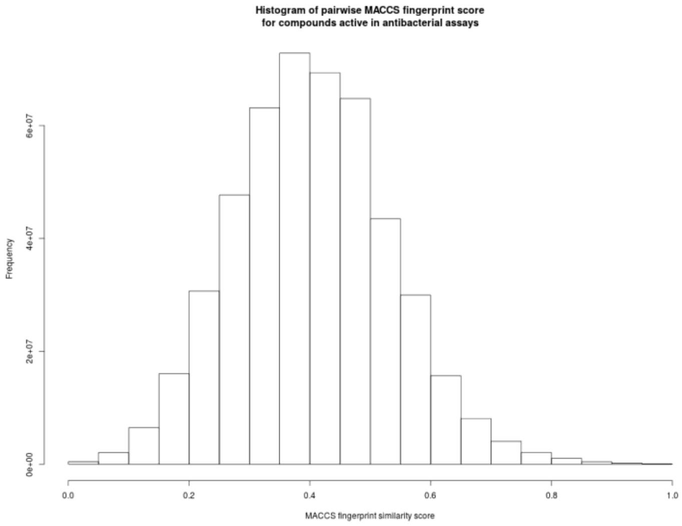

c)

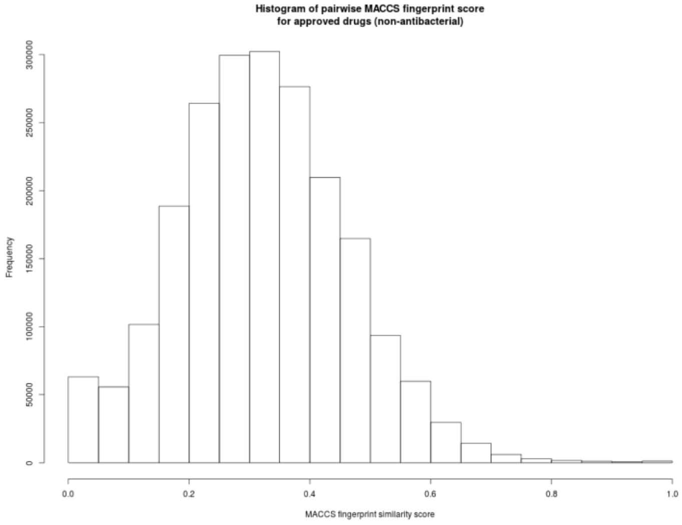

Figure S2. Boxplot for time series of molecular weight of antibacterial active compounds in the ChEMBL dataset. Any AA compound with an antibacterial activity record from a publication in that year is included.

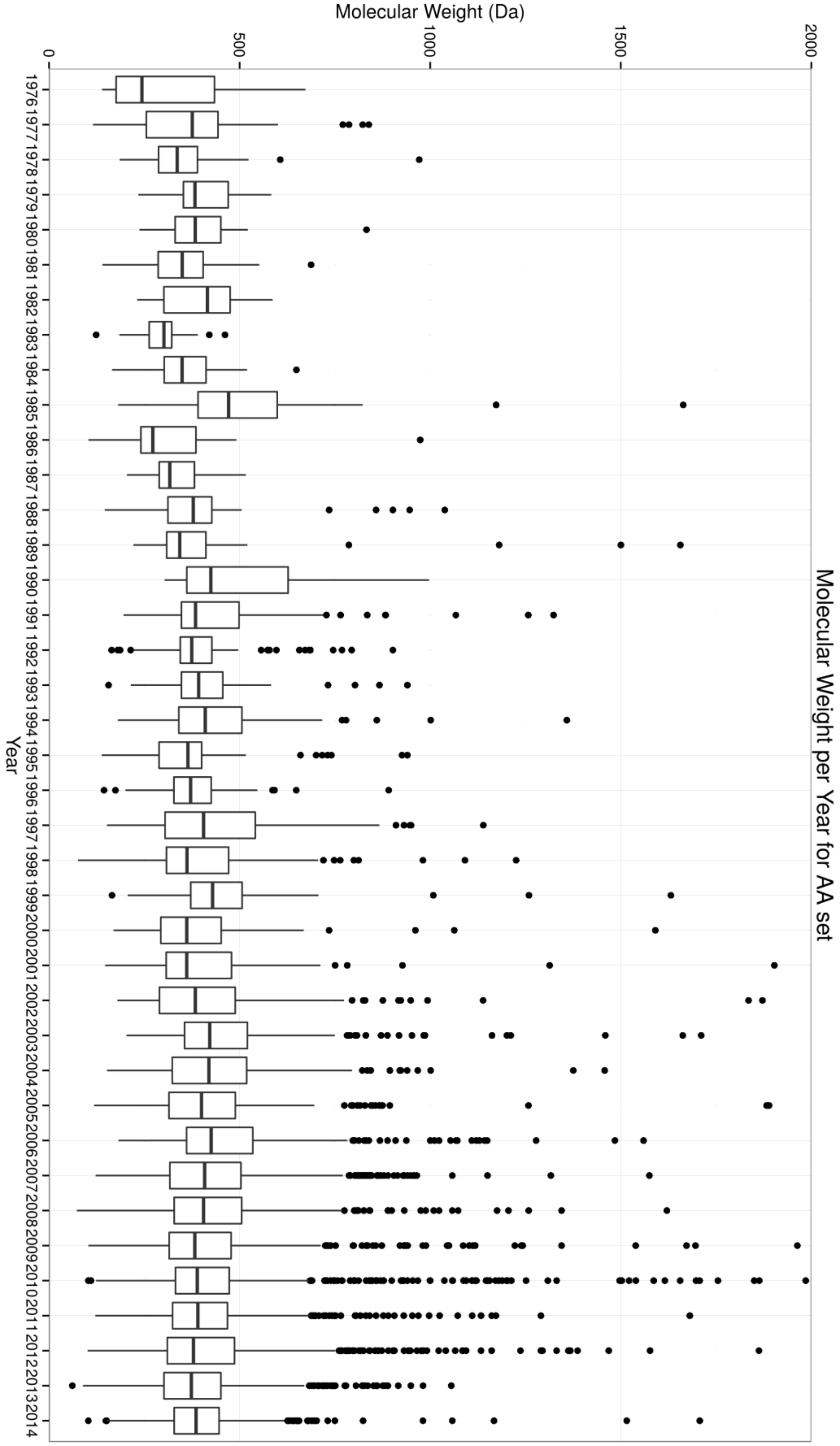

Figure S3. Boxplot for time series of calculated logP of antibacterial active compounds in the ChEMBL dataset. Any AA compound with an antibacterial activity record from a publication in that year is included.

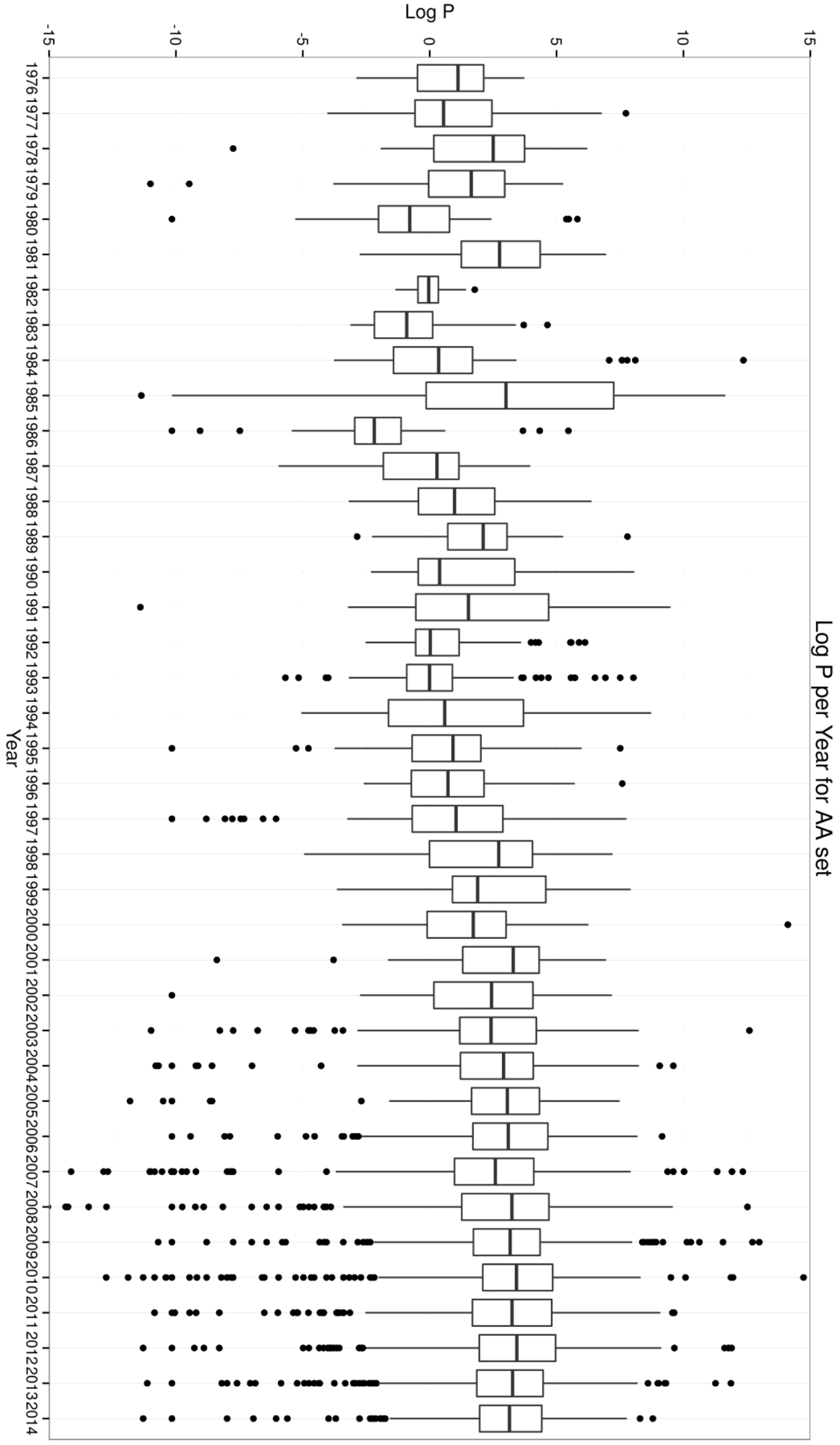

Figure S4. Property distribution comparisons for Gram negative and Gram positive active compounds.

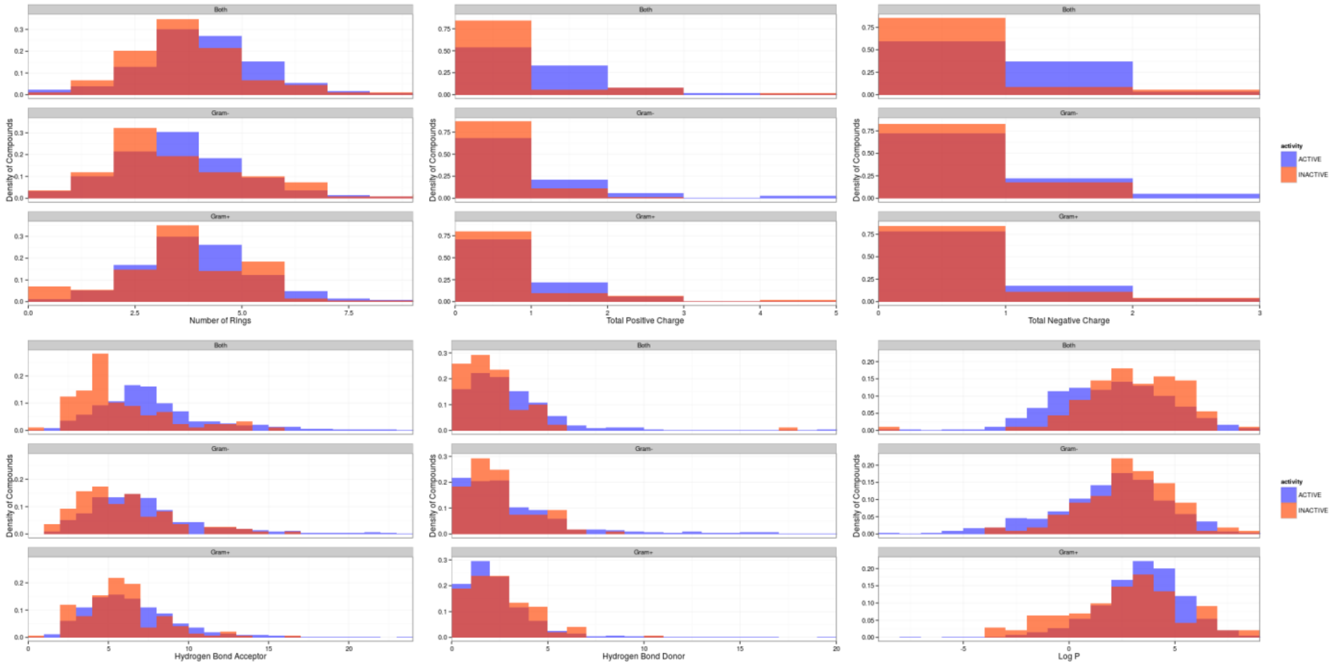

Supplement: Supplementary file 1 — 10.1186/s13321-016-0143-5 Table S1. Filters used to extract compounds from Antibacterial assays. Table S2. Filters used to extract compounds from Biochemical assays. Table S3a. Filters used to define antibacterial and biochemical compounds as ACTIVE. Table S3b. Additional filters (only one is applied per activity) used to define antibacterial and biochemical compounds as ACTIVE. Table S4a. Filters used to define antibacterial and biochemical compounds as SLIGHTLY_ACTIVE. Table S4b. Additional filters on the 'activities' table used to define antibacterial and biochemical compounds as SLIGHTLY_ACTIVE. Table S5. Filters used to define antibacterial and biochemical compounds as INACTIVE. Table S6. Filters used to extract compounds which have been approved as drugs. Table S7. Computed Descriptors for Dataset. Figure S1. Pairwise Tanimoto similarity of the MAD (a), AA (b) and MOD (c)sets. These distributions were calculated on the full sets, prior to clustering. Figure S2. Boxplot for time series of molecular weight of antibacterial active compounds in the ChEMBL dataset. Figure S3. Boxplot for time series of calculated logP of antibacterial active compounds in the ChEMBL dataset. Figure S4. Property distribution comparisons for Gram negative and Gram positive active compounds. [file 13321_2016_143_MOESM1_ESM.pdf]
